# Toward a mechanistic understanding of trophic structure: inferences from simulating stable isotope ratios

**DOI:** 10.1007/s00227-018-3405-0

**Published:** 2018-08-23

**Authors:** Kevin J. Flynn, Aditee Mitra, Antonio Bode

**Affiliations:** 1 Biosciences, Swansea University, Singleton Park, Swansea, SA2 8PP UK; 2 Instituto Español de Oceanografía (IEO), Centro Oceanográfico de A Coruña, Apdo. 130, 15080 A Coruña, Spain

## Abstract

Stable isotope ratios (SIR) are widely used to estimate food-web trophic levels (TLs). We built systems dynamic N-biomass-based models of different levels of complexity, containing explicit descriptions of isotope fractionation and of trophic level. The values of δ^15^N and TLs, as independent and emergent properties, were used to test the potential for the SIR of nutrients, primary producers, consumers, and detritus to align with food-web TLs. Our analysis shows that there is no universal relationship between TL and δ^15^N that permits a robust prognostic tool for configuration of food webs even if all system components can be reliably analysed. The predictive capability is confounded by prior dietary preference, intra-guild predation and recycling of biomass through detritus. These matters affect the dynamics of both the TLs and SIR. While SIR data alone have poor explanatory power, they would be valuable for validating the construction and functioning of dynamic models. This requires construction of coupled system dynamic models that describe bulk elemental distribution with an explicit description of isotope discriminations within and amongst functional groups and nutrient pools, as used here. Only adequately configured models would be able to explain both the bulk elemental distributions and the SIR data. Such an approach would provide a powerful test of the whole model, integrating changing abiotic and biotic events across time and space.

**Electronic supplementary material:**

The online version of this article (10.1007/s00227-018-3405-0) contains supplementary material, which is available to authorized users.

## Introduction

Accurate estimation of the trophic levels (TL) occupied by functional groups or specific organisms is often considered an essential requirement for the determination of the food-web structures. Historically, TLs were assigned through analysis of consumer diet composition (e.g., Pauly et al. 1998; Araujo et al. 2011). However, this approach is confounded by daily changes in diet with resource (prey, food) availability, and by difficulties in analysing gut contents (e.g., Cépède 1907; Conway et al. 1998). An alternative method for the determination of TLs makes use of natural abundance stable isotope ratios (SIR) of key elements (e.g., as ^15^N/^14^N or, less frequently, ^13^C/^12^C) (e.g., Boecklen et al. 2011; Layman et al. 2012).

The concept behind the use of SIR is that the TL is reflected by the cumulative isotopic transformations occurring at key biochemical conversions (Cabana and Rasmussen 1996; Robinson 2001; Post 2002; Fry 2006; Jennings et al. 2008). Values of the ratio between heavy and light isotopes of C or N within biochemicals or whole organisms are reported as transforms relative to the isotope ratio in a standard using the δ notation; for N, atmospheric N_2_ is the standard, being accorded δ^15^N = 0. The organismal value of δ^15^N represents a balance of assimilatory and dissimilatory processes throughout the food chain. The lighter isotope (here, ^14^N) is processed more rapidly; so, primary producers assimilate ^14^N-nitrate and ^14^N-ammonium more quickly than they do their ^15^N-nutrient counterparts, with the organisms becoming isotopically lighter (δ^15^N declines) relative to the source inorganic N. A similar fractionation occurs during ammonium regeneration by consumers, and so they become progressively isotopically heavier (δ^15^N increases). The cumulative fractionation at each and every biochemical step is ultimately expressed as an emergent property of whole organism isotope fractionation (Δ^15^N), with progressive organismal isotopic enrichment up through the consumer chain.

Empirical evidence indicates that isotope composition varies markedly among organisms within different TLs. An average difference in δ^15^N of 3.4‰ between adjacent TLs has been suggested to be remarkably constant among different types of consumers (Minagawa and Wada 1984; Van der Zanden and Rasmussen 1999, 2001; Post 2002). This has been ascribed to commonality in the net isotopic fractionation in biochemical reactions and physiological processes throughout the food web (Fry 2006). Accordingly, knowledge of the δ^15^N values of a given consumer and of a reference TL near the base of the food web has been proposed to enable estimation of the consumer TL by applying a constant isotopic enrichment (Cabana and Rasmussen 1996; Post 2002).

The SIR approach fosters an appreciation of the complexity of food webs by providing TL estimations for a variety of consumers (e.g., Bode et al. 2007; Agersted et al. 2014), and how TL may change under different conditions (e.g., Boersma et al. 2016). However, the assumption of a constant enrichment between TLs represents a potential major source of error in the estimations (Caut et al. 2009; Hussey et al. 2014; Jennings and van der Molen 2015). The concept of relating SIR to TL has also been questioned (e.g., Layman et al. 2007 versus Hoeinghaus and Zeug 2008).

Variability around mean isotopic enrichment values between and within different species (Van der Zanden and Rasmussen 2001; McCutchan et al. 2003; Caut et al. 2009; Hussey et al. 2014) are related largely to changes in diet (Dijkstra et al. 2008), and temporal variations in the inorganic N source for primary producers (Deudero et al. 2004; Bodin et al. 2007; Jennings et al. 2008; Matthews and Mazumder 2005). Consumers feeding upon organisms of lower trophic levels (notably upon herbivores) often display lower isotopic N enrichment than the average for the food web (Van der Zanden and Rasmussen 1999, 2001; Matthews and Mazumder 2008); the same has been noted for top predators (Hussey et al. 2014), and also for parasites (Pinnegar et al. 2001; Persson et al. 2007). Consumption of detritus, much originating from primary consumers, together with opportunistic omnivory, also affects the low enrichment observed between organisms (e.g., in planktonic systems—Rau et al. 1990; Rolff 2000; Bode et al. 2003; Matthews and Mazumder 2008). Further, abiotic stress can affect trophic discrimination in generalist consumers (Reddin et al. 2017). Uncertainties in δ^15^N at the base of the food web and in trophic fractionations then propagate, causing uncertainty in assignment of TLs (Jennings and van der Molen 2015). The fact remains, however, that SIR provides the only tool that integrates organism activities over a meaningful period of their life span.

Determining the exact TL and SIR over a time course of trophic interactions in nature, as required to rigorously test the relationship between the two, is not possible. The test also needs to be conducted for food webs of different complexity and dynamics. Here, we explore the utility of the SIR approach as a predictor of TL through the use of a system dynamics modelling approach in a way that is not possible empirically. To achieve this, we have taken a dynamic model of a food web operating with a parallel explicit description of isotope discrimination, and compared the SIR signature against the computed real TL over a prolonged simulation period with frequent data sampling. We repeated the analysis using food webs of different levels of complexity. As an exemplar, we used as our study system variants of the widely used N-based marine plankton food web (Fasham et al. 1990). The main food-web model comprised nutrient sources, phytoplanktonic primary producer, four zooplanktonic consumers of increasing size, and also detritus.

## Materials and methods

### Model food-web description

**Fig. 1:**
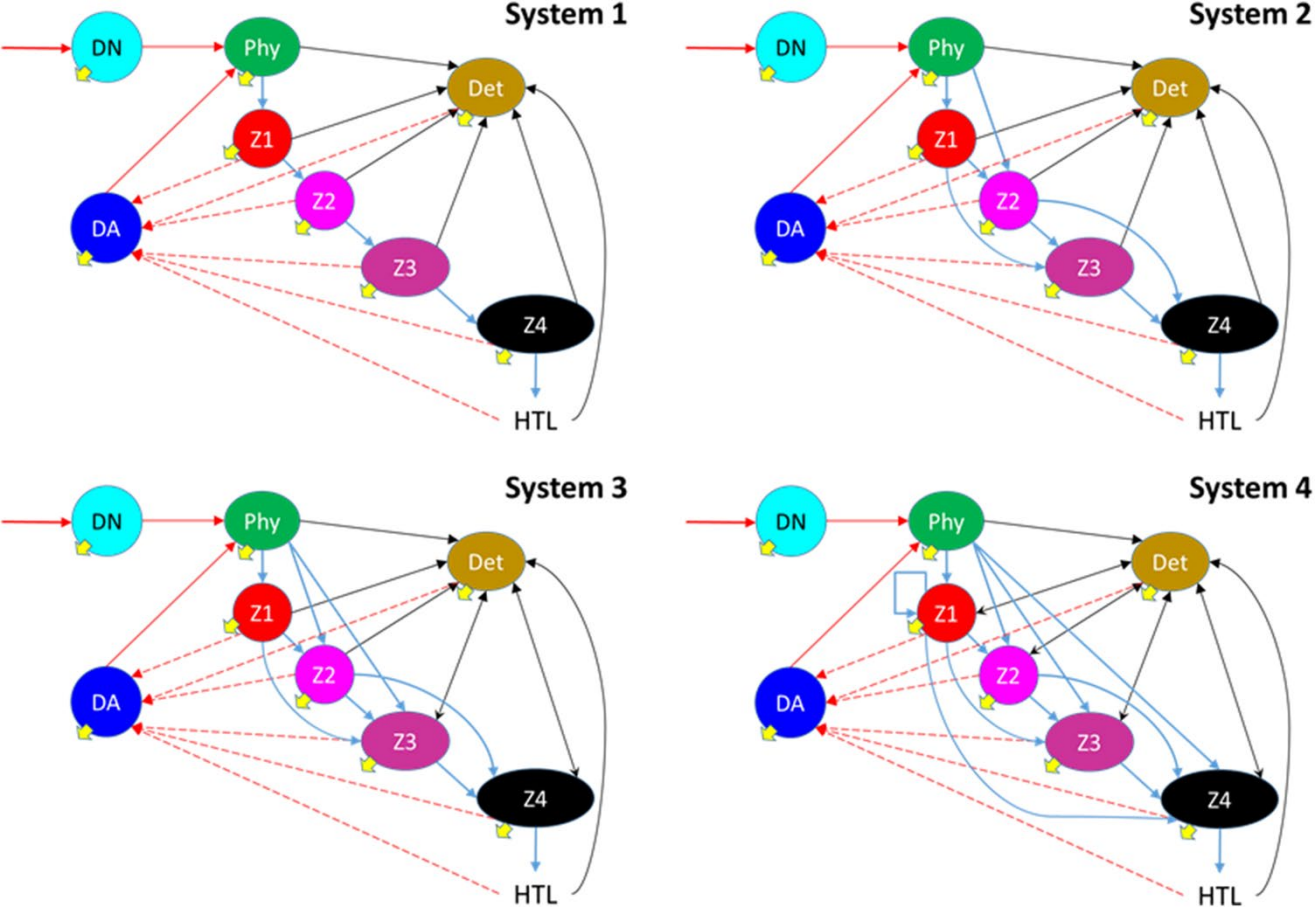
Food-web structures used in the simulations. Phytoplankton (Phy) uses nitrate (DN) brought into the system and (by priority) ammonium (DA) which is regenerated within it. Nitrate enters the photic zone via mixing (at a rate of 0.05 day^−1^) from below the thermocline and the same mixing removes all components (yellow arrows); in essence, the system works akin to a chemostat with nitrate as the only nitrogenous component in the in-flow. All zooplanktonic consumers (Z1–Z4) void a proportion of their food, which then contributes to the detritus pool (Det), and also release ammonium. The detritus pool decomposes to contribute to the ammonium pool. Z4 is also grazed by higher trophic levels (HTL). Note some of the arrows linked to detritus are double-headed, and that Z1 in System 4 can cannibalise. The colours used for the nutrients, organisms and detritus are those used to identify data series in Figs. 2, 3, 4, and 5

A detailed description of the model is given in electronic supplementary materials (ESM), online. A N-based model was constructed describing a 5-level functional type (FT) planktonic system, where one FT (*Phy*) was the primary producer (assumed as non-mixotrophic phytoplankton, and hence assigned TL = 1), together with 4 FTs assigned as consumers (here identified as zooplankton *Z1* to *Z4*). These FTs were interconnected in different ways to provide a series of food webs of increasing complexity (Fig. 1). The activity of all FTs contributed to a common detrital pool; the death rate of *Phy* increased with deteriorating N-status thus providing phyto-detritus, while *Z1*–*Z4* contributed to detritus through release of unassimilated (voided) ingestate, as well as via their own death (increasing with deteriorating nutrient status).

Food selection and consumption were described using the approaches of Mitra and Flynn (2006) and Flynn and Mitra (2016), taking into account the likelihood of prey encounter according to allometric constraints linked to motility, consumer and food sizes. *Phy* was assigned as a motile protist of equivalent spherical diameter (ESD) 10 µm, *Z1* was assigned as a 50 µm ESD protist microzooplankton, while *Z2*–*Z4* were assigned as mesozooplankton of ESDs 200, 500 and 2000 µm, respectively. Detritus was considered of similar biomass density as protists, and with a size equating to an ESD of 50 µm. The model was configured to enable the uppermost consumer, *Z4*, to be grazed by deploying a closure function that represents the activity of higher trophic levels (*HTL*). In the results shown here, however, it was not necessary to invoke this activity; doing so resulted in the extinction of Z4 within the 100+ day simulation period.

Values of constants defining the assimilation efficiency (AE) and maximum growth rates were selected so as to reflect typical literature values for plankton and provide a system that described cycles of trophic dynamics. As any changes in such dynamics affect both the TL status and the distribution of ^15^N/^14^N through the same interactions, the exact choice of these values of AE, respiration and growth rates does not affect the general interpretation of our results.

Entry and exit of nutrients and biomass are important factors affecting ecology and also the dynamics of stable isotope distribution. In our modelled planktonic system, entry and exit were described as the process of mixing between the photic and sub-photic zones (set here at a rate of 0.05 day^−1^). This mixing introduced new nitrate and removed a fraction of all residual nutrients, biomass and detritus (i.e., described explicitly as total N and as ^15^N, and implicitly also ^14^N) from the photic zone.

### Isotope sub-model

To the description of the fluxes of total *N* around the food web, we added a parallel model describing the flow of ^15^N, with differences in N-specific flow rates accounted for by isotope fractionation. Isotopic fractionation is defined by *α*; values of *α* > 1 indicate fractionation (typical values of *α* are ca. 1.005–1.05; Wada and Hattori 1991), while *α* = 1 indicates no fractionation. Values of *α* for different physiological processes were taken as mid-point estimates from Robinson (2001); the exact values of *α* within the plausible range reported in the literature does not affect the conclusions drawn in our work.

Abiotic mixing process exerted no isotope fractionation but brought in nitrate with δ^15^N = 5‰ (Pantoja et al. 2002). N-source selection by *Phy* assumed priority consumption of the ammonium regenerated by the consumers; any shortfall in provision of nutrient-N was then “topped off” by consumption of the nitrate. Values of *α*_nitrate_ and *α*_ammonium_ were set at 1.009 and 1.015, respectively.

No isotope fractionation was applied at food selection. Within each consumer, food (prey and/or detritus) was assumed to be processed collectively with the same assimilation efficiency (AE; the balance, as 1-AE, being voided) and with no isotopic discrimination. Within the feeding vacuole or gut, the dynamics of assimilation from the digestate were considered akin to a sealed system and hence δ^15^N_digestate_ was considered to be the same as δ^15^N_food_, with *α*_void_ = 1. In reality, some level of differential assimilation and isotopic discrimination could occur, driven by digestive enzyme activity and metabolite transport across the vacuole membrane or gut wall. This could depend on the duration of digestion set against variable gut transit time (Mitra and Flynn 2007).

Assimilated material may remain within the consumer (contributing to somatic growth) or be respired. Respiration is directly associated with anabolic processes (assimilation and growth with specific dynamic action), and to catabolic respiration (including that associated with homeostasis). Respiration, and the allied regeneration of ammonium, incurs isotope fractionation. Anabolic respiration and regeneration act primarily on the isotopic composition of the incoming digestate, while catabolic respiration and regeneration (especially for N) act primarily upon the isotopic composition of the body. Isotopic fractionation of ^13^C/^12^C is affected by such processes through the differential deposition of C in proteins versus lipids (De Niro and Epstein 1977; see also Wolf et al. 2009); this sets the basis of the work by Pecquerie et al. (2010), exploring isotope fractionation via energy flows in consumers. In our model, as N is primarily apportioned to structural moieties, N isotope fractionation was applied equally to anabolic and catabolic events, with *α*_regeneration_ = 1.006.

The isotopic signature of the detritus varied as a function of the isotopic composition of the voided material that contributed to it and activities that then degraded it. Release of N associated with the consumption of *Z4* by *HTL*, as described by the closure term, and with the degradation of detritus, was returned to the inorganic nutrient pool, as ammonium. This process was considered to be complete within the photic zone and accordingly the isotopic composition of the ammonium flux reflected the isotopic content of the source material, with no isotopic fractionation.

### Trophic level sub-model

TLs were not apportioned through reference to the web structures shown in Fig. 1; the value of the TL for each FT was computed through reference to the dynamics of N flowing through the biotic system. While *Phy* was confined to TL = 1, the status of the other components (*Z1*, *Z2*, *Z3*, *Z4*, detritus) each changed as a function of their prior TL, and with the TL status of the incoming contributing nitrogenous material.

A special instance in allocation of the TL was that for the detrital pool. The importance of the detrital pool and activities of its components in ecology is often understated (Moore et al. 2004), or “invisible” (Gutiérrez-Rodríguez et al. 2014), with the exact origins of the material (including allochthonous sources) causing additional challenges in SIR interpretation (Mallela and Harrod 2008; Nilsen et al. 2008; Docmac et al. 2017). In reality, a proportion of what is in the modelled detrital material would be microbial detritivores. To fully describe this material would require explicit description of the microbial consortia and of their activities; this was considered to be beyond the needs of the current work. While the TL status of detrital material including any allied microbes would be higher than indicated here, as both the calculated TL and δ^15^N values were subjected to the same assumptions, the validity of our test is maintained.

### Simulations

We present the results from explorations of four food-web configurations (Fig. 1), with increasing levels of complexity from a simple linear food chain (System 1) to the most complex (System 4), in which each consumer could feed on detritus and on primary producers, and *Z1* (here considered as a protist microzooplankton) could also cannibalise. Simulations were run at three input nitrate concentrations. Values of δ^15^N for different components are reported using the syntax δ^15^N_component_.

Understanding the dynamics of δ^15^N is complicated by the combination of dilution and discrimination events operating over the time prior to sampling (Robinson 2001; Phillips et al. 2014). Models also require a period of spin-up to establish the values of state variables for TL and δ^15^N. Accordingly, sampling the simulation data was commenced 20 or more days after the start of simulations.

## Results

**Fig. 2:**
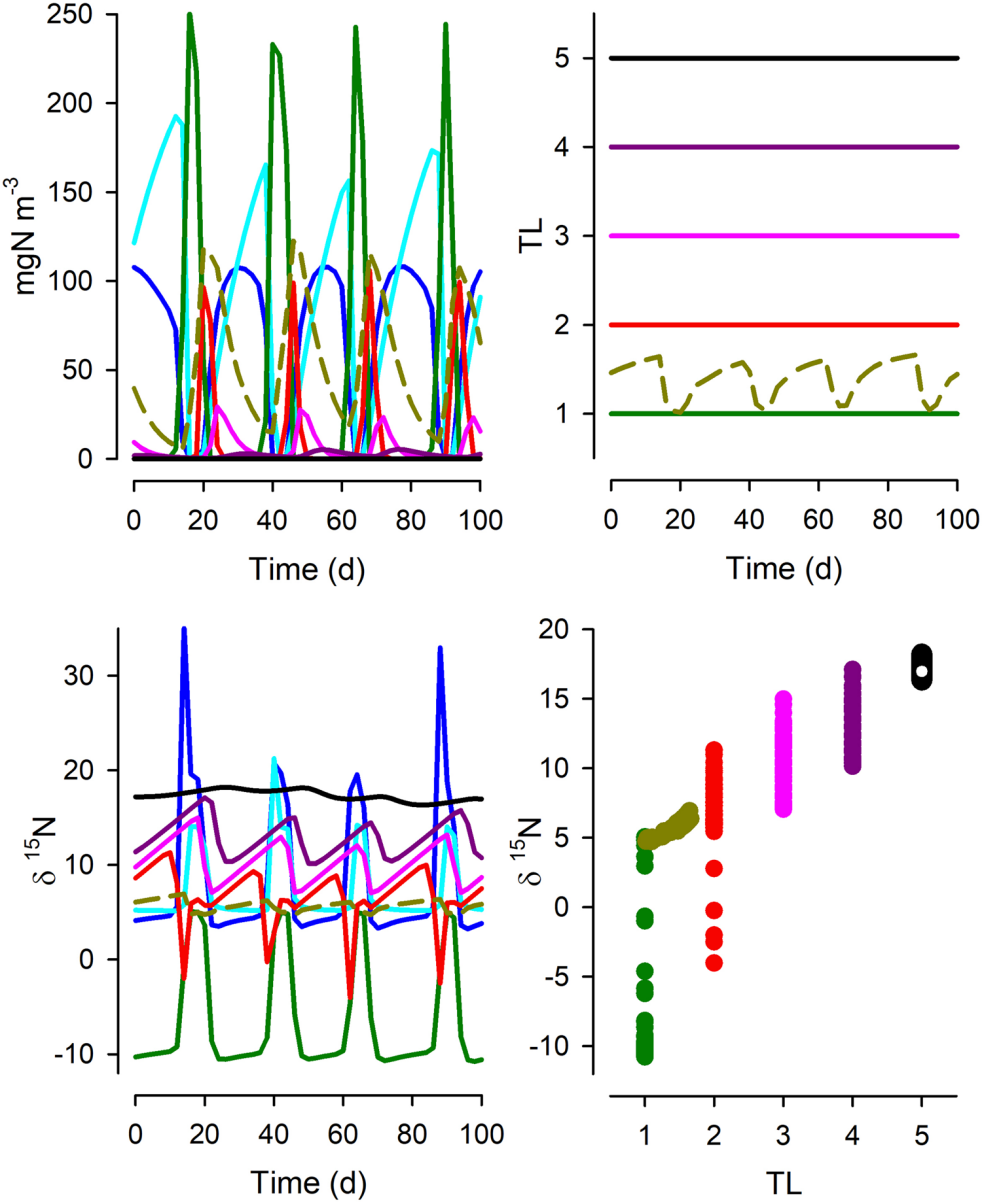
Time plots of the partitioning of N in System 1 between dissolved nitrate (DN; cyan), ammonium (DA; blue), phytoplankton (Phy; green), four zooplankton functional types (Z1–Z4; red, pink, dark pink and black, respectively) and detritus (brown). Other time plots show the values of δ^15^N and trophic level (TL) of the biotic components. See Fig. 1 for details of the food web structure. The relationship between TL and δ^15^N is shown for each functional group; the slope of the relationship is given in Table 1 as the “midN” value

**Fig. 3:**
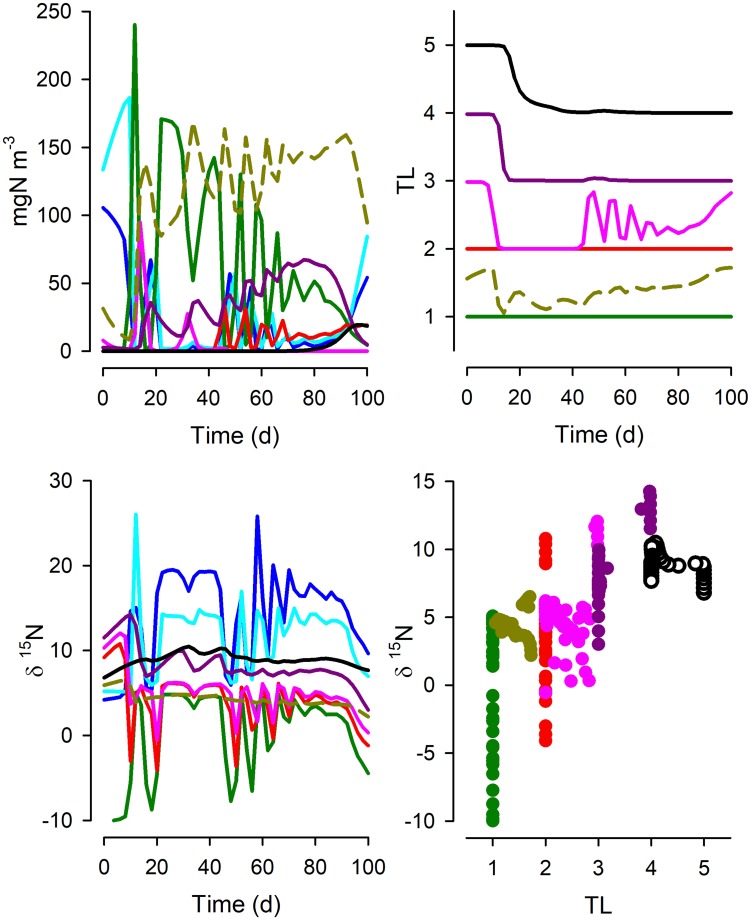
As Fig. 2 but for System 2; see legend of Fig. 2 for an explanation of line and symbol types. See also Fig. 1 and Table 1

**Fig. 4:**
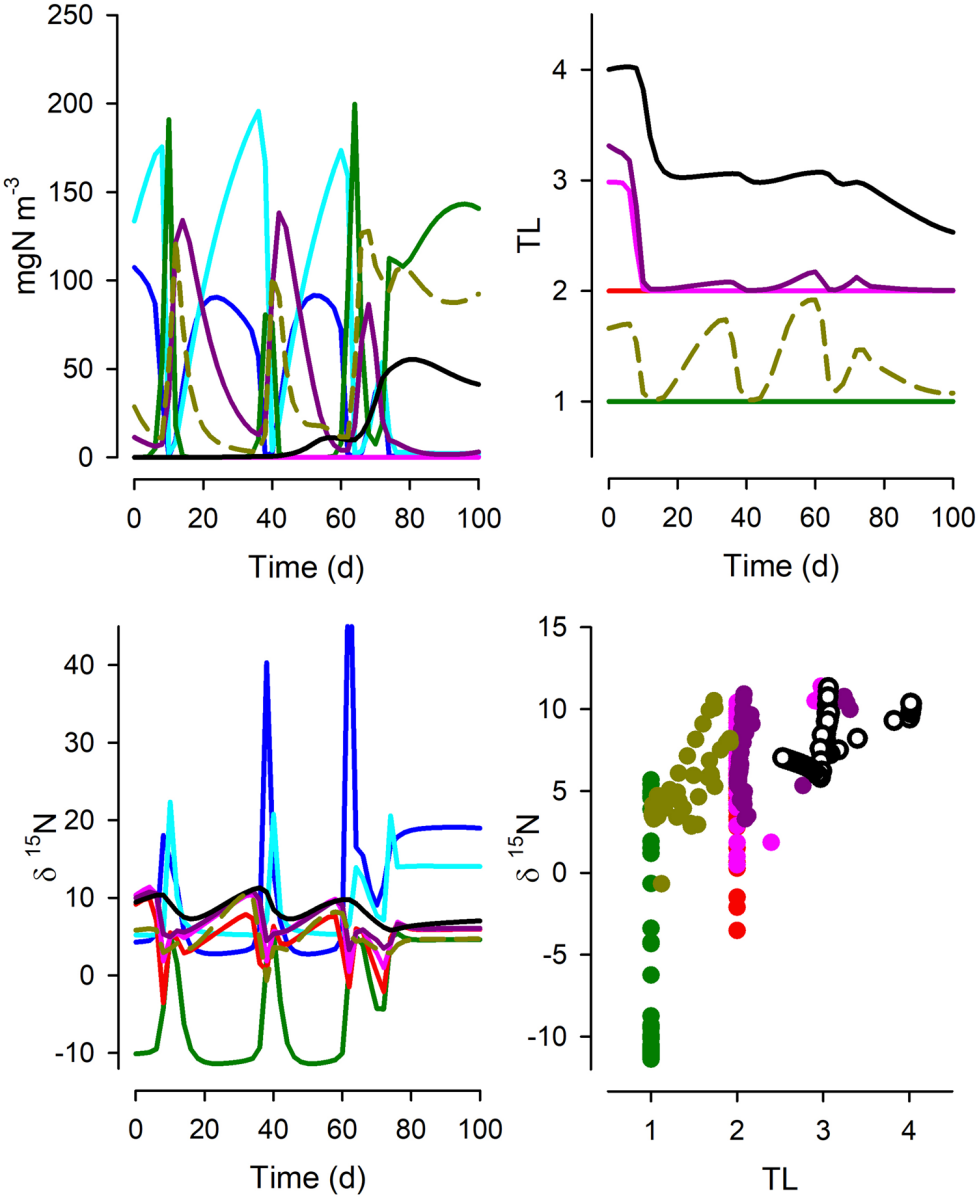
As Fig. 2 but for System 3; see legend of Fig. 2 for an explanation of line and symbol types. See also Fig. 1 and Table 1

**Fig. 5:**
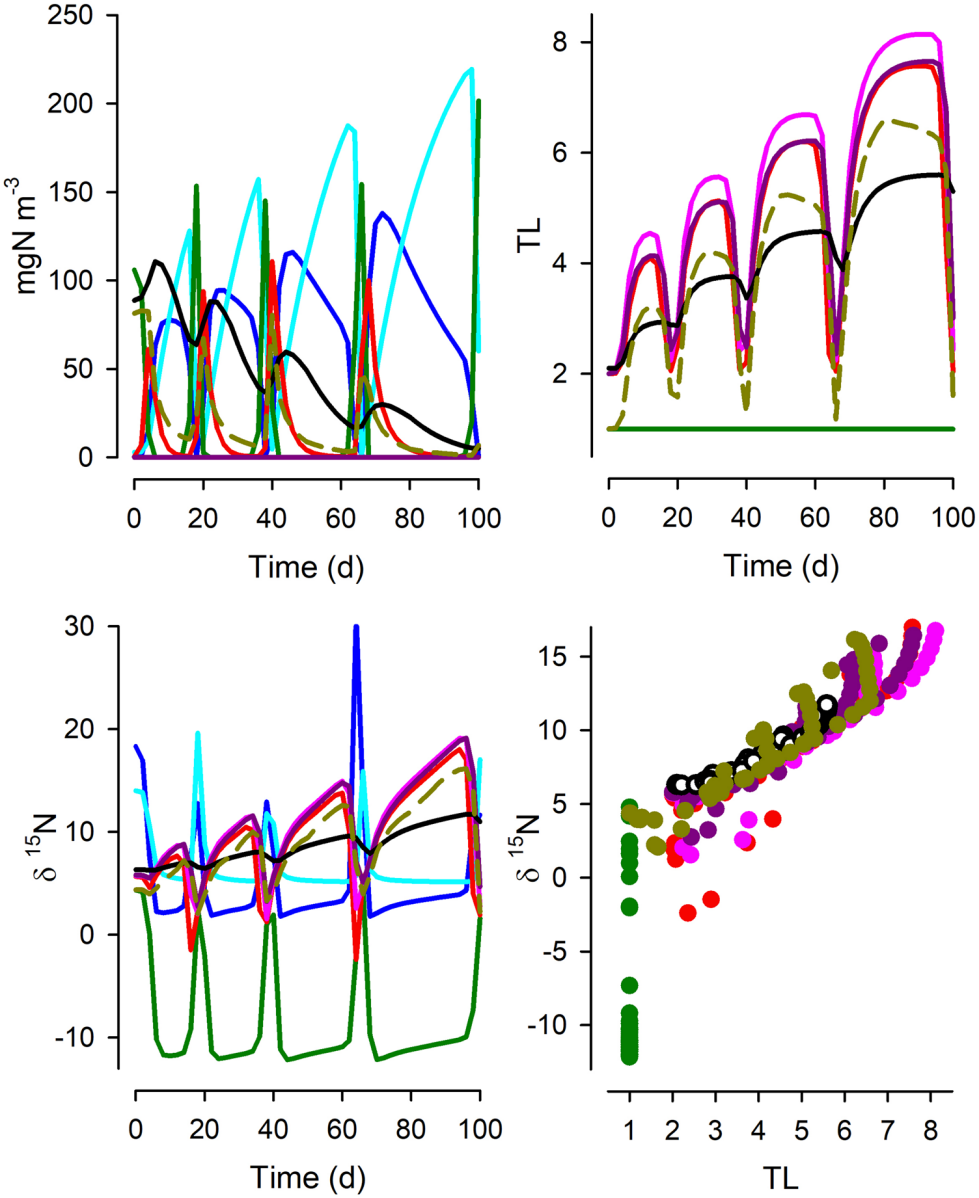
As Fig. 2 but for System 4; see legend of Fig. 2 for an explanation of line and symbol types. See also Fig. 1 and Table 1

**Fig. 6:**
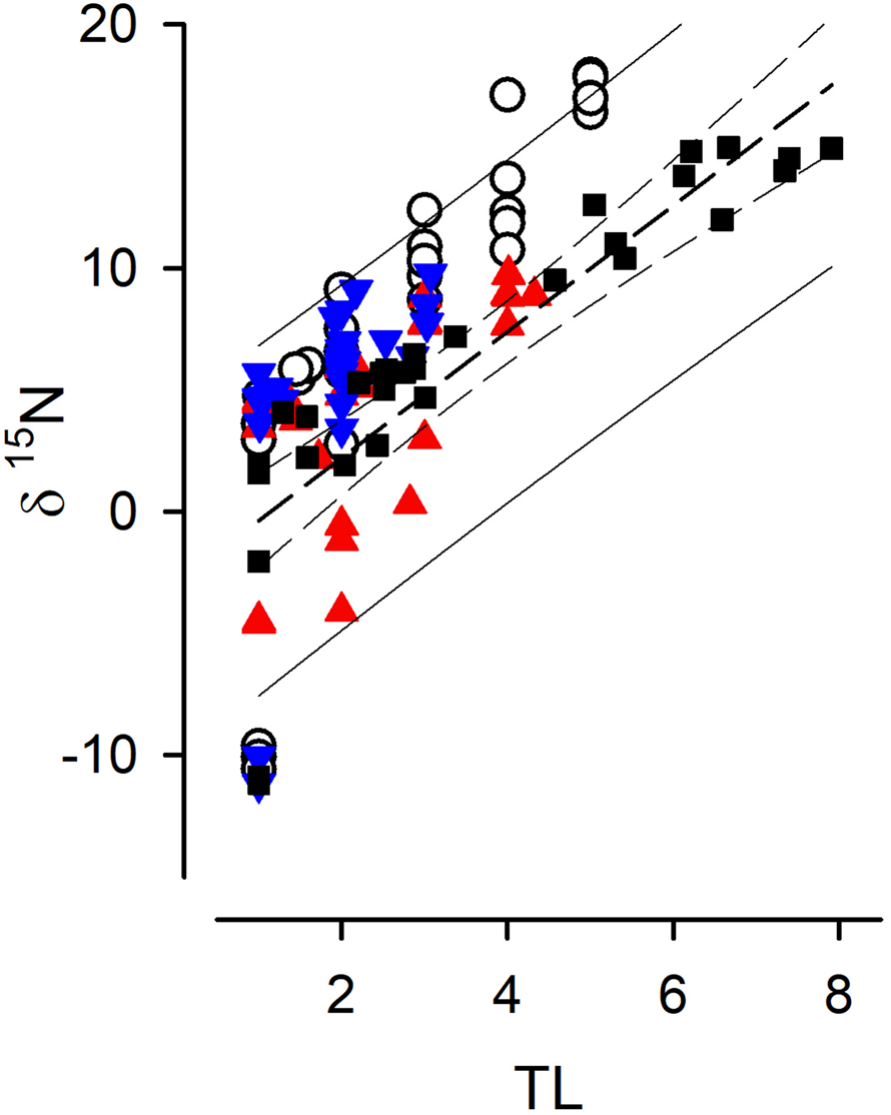
The relationship between TL and δ^15^N for data taken every 20 days from the simulations shown in Figs. 2, 3, 4 and 5. System 1—open circles; System 2—red triangles; System 3—blue inverted triangles; System 4—black squares. See also Table 1. The dark dashed line is the line of best fit (linear regression) through all the plotted data, the thin dashed lines are the 95% confidence limits for that best fit, and the thin continuous lines are the 95% predictive limits (computed by SigmaPlot 12.5, Systat Software Inc)

Figures 2, 3, 4, 5 and 6 show results for the mid-level nitrate input (20 µM), while summary statistics for all systems and nitrate inputs are shown in Table 1.

**Table 1:** Relationship between trophic level (TL) and δ^15^N for simulations run within food webs of different complexity (see Fig. 1)

Data sampled	System	loN		midN		hiN	
		Slope	*R* ^2^	Slope	*R* ^2^	Slope	*R* ^2^
All	1	2.68	0.90	4.72	0.70	6.61	0.76
	2	2.03	0.70	2.50	0.45	3.76	0.48
	3	1.97	0.35	4.61	0.38	7.51	0.46
	4	2.30	0.41	3.28	0.77	3.05	0.36
	All			2.78	0.53		
All except Phy	1	2.79	0.91	3.24	0.83	5.28	0.78
	2	1.96	0.72	1.95	0.44	2.57	0.47
	3	1.75	0.29	2.18	0.30	2.11	0.10
	4	1.56	0.50	2.14	0.86	1.30	0.22
	All			2.16	0.61		
All except Phy & detritus	1	3.03	0.91	3.48	0.77	5.45	0.68
	2	2.03	0.64	2.21	0.41	2.46	0.35
	3	1.59	0.16	2.39	0.25	1.18	0.03
	4	1.47	0.44	2.19	0.87	1.23	0.19
	All			2.30	0.57		

These relationships considered model outputs taken every 2 days over a 100 day period following a spin-up period of at least 20 days. The data shown in Figs. 2, 3, 4, 5 and 6 were from systems supplied with 20 µM nitrate, labelled “midN” here. Simulations were also run at 10 µM nitrate (“loN”) and 40 µM nitrate (“hiN”). Also, shown are the relationships for the entire data series shown in Fig. 6 (system “all”; “midN” only). The relationship (slope) was calculated either with inclusion of all organisms and detritus (“All”), excluding the phytoplankton data (“All except Phy”), or excluding phytoplankton and detritus (“All except Phy & detritus”; i.e., only considering consumers *Z1*… *Z4*). These slopes may be compared to the proposed expected value of δ^15^N = 3.4‰ per TL (Minagawa and Wada 1984; Van der Zanden and Rasmussen 1999, 2001; Post 2002)

Figure 2 shows the functioning of the simplest system, as a linear food chain (Fig. 1, System 1). In the presence of sufficient nitrate, the discrimination at N-source assimilation during initial primary production gives a strongly negative δ^15^N_Phy_. During bloom development, as N sources become limiting, both the residual nutrients (δ^15^N) and δ^15^N_Phy_ become increasingly positive. At the peak of the blooms, as almost all ^14^N and ^15^N nitrate becomes assimilated into *Phy*, δ^15^N_Phy_ becomes similar to that of the incoming nitrate (δ^15^N_nitrate_ = 5‰). These transients in δ^15^N_Phy_ feed into the consumer chain and, although the trophic levels (TL) remain constant for each functional type in this simple linear food chain (except for detritus), the value of δ^15^N for each component varies greatly with the cycles of predator–prey dynamics. In consequence, the relationship between TL and δ^15^N shows considerable spread. Table 1 shows that the slope of the relationship, either with reference to all biomass groups, excluding phytoplankton, or excluding phytoplankton and also detritus. Even for this simplest of systems, the slope of the relationship in the simulation output is not constant, though the value of *R*^2^ is high. Further, the slope varies as a function of the resource abundance (altered here by lower and higher nitrate input levels, noting that these concentrations affect consumer abundance and thence also predator–prey activities); the slope of TL vs δ^15^N was higher when the system operated at higher resource abundance.

System 2 differs from System 1 by having additional predator–prey interactions (Fig. 1). The dynamics are very different (Fig. 3, cf. Figure 2), and the trophic level of individual consumer types now varies over the simulation period. The relationship between TL and δ^15^N is poorer than for System 1 and the slopes are notably lower (Table 1). System 3 sees additional food-web complexity, including now an ability by Z3 and Z4 to consume material from the detrital pool (Fig. 1). In this system, Z2 and Z3 become minor components, so that Z4 de facto acquires a much lower TL than may be expected from the web construction (Fig. 4). The relationship between TL and δ^15^N differs further from those for Systems 1 and 2 (Table 1), again with the “All” data relationship returning a higher TL vs δ^15^N slope from high-resourced systems.

System 4 is the most complex food web, including cannibalism (intraguild predation) within *Z1*, with a wider range of food options and all zooplankton capable of consuming detrital material (Fig. 1). Here, the TL of each plankton FT changes greatly over the food-web cycles (Fig. 5), and over time the TLs also increase due to the combined effects of cannibalism and recycling of detrital material. Despite this, the relationship between TL and δ^15^N is in keeping with those seen in the other simulations, though again varying depending on the resource abundance (Table 1).

Figure 6 shows the relationship between TL and δ^15^N from samples taken, at 20-day intervals after the initial spin-up period, from all systems run at the mid-nitrate abundance (as per Figs. 2, 3, 4, 5). Table 1 gives the slopes of the relationship across all of these systems, showing (as with the systems considered individually) a steeper slope when all functional types (*Phy*, *Z1*… *Z4* and detritus) are included, less steep when only the consumers (*Z1*… *Z4*) are considered, and a lower slope again when considering consumers plus detritus excluding just the phytoplankton.

## Discussion

The purpose of establishing the trophic level (TL) of different groups of organisms is to aid the understanding of ecological linkages, supporting the construction of conceptual and thence computational descriptions of food webs. The use of changes in values of δ^15^N and δ^13^C, in theory, provides a method to determine trophic activity integrated over time and space. There are, however, clear challenges in decoding stable isotope signatures in field and also laboratory microcosms (Farquhar et al. 1989; Flynn and Davidson 1993; Auerswald et al. 2010; Layman et al. 2012), many of which centre around issues of isotope discrimination at different biochemical and trophic levels, of isotope cycling and dilution (Robinson 2001). While alternate nitrogen pathways may be indicated by consumer δ^15^N time series data (Matthews and Mazumder 2007), multiple system components need to be examined to assess trophic structure in complex food webs. Phillips et al. (2014) give recommendations on modelling approaches exploiting SIR data, stressing the importance of suitable spatial–temporal and organism sampling strategies. Though easier to follow for some food webs than for others, these recommendations are invariably challenging. Field sampling is typically extremely patchy in time and space, and especially for microbial systems (e.g., plankton) simply separating different functional groups for analysis can be very difficult.

What our work shows is that even in the utopian situation of having a full data set from a well-studied system, we cannot expect a simple robust relationship between δ^15^N and TL. The same food-web structure, but just operating under different nutrient loads, is seen to exhibit different relationships. This is demonstrated by the variation in TL vs δ^15^N slope values when we simulated the food webs under different resource availabilities (Table 1). The totality of the complexity of unravelling isotope discrimination relative to TL is additionally demonstrated by Figs. 2, 3, 4, 5 and 6. While we may be able to explain changes in δ^15^N through reference to our understanding of ecophysiology and ecology, the converse (using δ^15^N as an explanatory tool) appears challenging even if we have an ideal series of SIR data.

### The failure of SIR to describe TLs

Models exploiting field data typically access SIR values at very few time points, and, thus, attempt to rebuild trophic dynamics from SIR values that are themselves affected by many, un-sampled, dynamic processes. Different modelling strategies have tried to make sense of the relationship between TL and δ^15^N; Middelburg (2014) gives an overview of methods used to make inferences about trophic structure from SIR. Models in this context include simple statistical approaches, through to Bayesian approaches and simulations (Parnell et al. 2010, 2013; Kadoya et al. 2012; Van Engeland et al. 2012; Brett et al. 2016; Yeakel et al. 2016). Inverse modelling approaches have also been proposed (Eldridge et al. 2005; Van Oevelen et al. 2010), to de facto rebuild the most likely trophic system that could explain the SIR data. Our approach contrasts with other modelling attempts to explore the relationship between SIR and TL, through explicitly modelling the flow of ^15^N, involving biochemical fractionation rather than implicit or assumed organismal fractionation, and also by calculating TLs.

A key challenge in this arena is that the relationship between TL and δ^15^N is dynamic and, because of various factors, each of these variables changes out of sequence with each other. Indeed, the timing sequence itself varies with the dynamics of the physiology and interconnectivity of the organisms, which is in turn affected by resource (nutrient, food, prey) availability. The dynamics are affected by the nutrient loading because high loading raises biomass levels and hence encounter rates, and allied predator–prey interactions change. Olive et al. (2003) specifically explored the dynamics of SIR change during diet switching making use of a linear model; this dynamic is portrayed in our model not only with respect to diet switching at one level, but with the subsequent cascade of SIR values (and changes in TL) through other parts of the trophic web. When considering isotope discrimination, there is an additional aspect related to nutrient loading, and that concerns the availability of ^14^N vs ^15^N in nitrate and ammonium, which then affects δ^15^N of the phytoplankton (e.g., see Fig. 2).

From our simulations, it can be seen that there is no universal relationship between TL and δ^15^N that permits a robust prognostic tool for configuration of food webs even if all system components can be reliably analysed. Thus, the slopes of the relationship between TL vs δ^15^N vary greatly between systems of different complexity and temporal dynamics and nutrient load (Table 1). This conclusion conflicts with suggestions that difference in δ^15^N of ca. 3.4‰ aligns with a difference of 1 trophic level (Van der Zanden and Rasmussen 2001). A consideration of a more dispersed sampling regime (Fig. 6), rather than tightly sequential sampling regime in systems of known history (Figs. 2, 3, 4, 5), also shows not only the spread in the slope but also the problem of establishing the intercept of the relationship. This affects the ability to determine the TL from a given SIR, as finding an appropriate baseline to make ecological inferences is far from trivial (Cabana and Rasmussen 1996; Post 2002; Matthews and Mazumder 2005); in nature the “baseline” can never be truly fixed.

An alternative approach to assuming a fixed TL- δ^15^N relationship is the development of empirical relationships to account for the decrease in δ^15^N enrichment up the food webs, as shown through meta-analysis of previous data for top marine predators (Hussey et al. 2014). In that way, the resulting food web structures have more TLs than would be estimated by traditional whole-ecosystem models based on constant ^15^N enrichment. The technique we deploy here could be used to check the power of such ideas from a conceptual basis. In our work, with the same number of functional groups though connected differently, 5 operational TLs emerge within System 1 (Fig. 2), while emergent TLs within System 4 exceeded 8 (Fig. 5). Furthermore, in System 4, where the protist *Z1* could be cannibalistic, the TL for *Z1* could exceed that for the mesozooplankton *Z4*; this reflects a series of predator–prey interactions within a functional grouping depending on the role of intraguild cannibalism and of detritivory. Thus, the colloquial interpretation of trophic levels (e.g., that larger metazoan have a higher TL than do micrograzers) can be seen to be challenged in food webs with a high level of dynamic complexity. The upward spiral in TL (Fig. 5) was matched by changes in δ^15^N such that the slope of the TL vs δ^15^N relationship was not dissimilar for System 4 as for other systems (Table 1).

The results of our simulations also help to explain the findings in field studies that show an uneven propagation of the δ^15^N through the food web (Bode et al. 2007; Jennings et al. 2008; Mompeán et al. 2013). Changes in the temporal variation in δ^15^N have been attributed primarily to body-size effects affecting the turnover of body tissues, as such changes are significantly greater in smaller animals (assumed of lower TL and higher specific growth rates) and decline continuously with body size (Jennings et al. 2008; Reum et al. 2015). Empirical studies also point to a key role of plankton size in determining the number of TLs (Hunt et al. 2015).

The effects of deploying different values of *α* at different stages can also be explored using the type of approach we used. Thus, the changing relationship between δ^15^N enrichment with organism size may relate to differences in discrimination at assimilatory (digestion and anabolism) versus dissimilatory (catabolic) processes relating to structure and biochemical/physiological differences between consumers, their rate and frequency of feeding, and their net growth rate. In our simulations, we set no discrimination at assimilation (*α*_void_ = 1), but alternative configurations could be readily explored.

### An alternative use for SIR data

Despite all efforts to provide a robust diagnostic tool for food-web studies using SIR (e.g., Layman et al. 2012; Jennings and van der Molen 2015), the combined complexities of the trophic dynamics, isotope discrimination and dilution appear to confound such usage. The rate at which the SIR in a given consumer approaches equilibrium is a function of the stability of the SIR in the diet and of the metabolic rate of the consumer (Woodland et al. 2012), a set of interactions that is repeated at each organism, and interacts throughout the ecosystem, as demonstrated in our simulations. It seems most likely that using SIR of specific biochemical markers (Brett et al. 2016) will be fraught with similar problems due to different levels of isotope discrimination at internal localised assimilatory and dissimilatory pathways.

Returning to the original purpose of why one wishes to know the TL, and how SIR signatures could aid in such determinations (i.e., integrating over temporal and spatial activities), it becomes apparent from our work that the ability of a dynamic ecosystem model to describe the SIR data through an explicit description of isotope fractionation dynamics, could itself provide a useful validation tool for systems ecology. This potential becomes even more powerful when considering the impacts of physical processes on the temporal and spatial distribution of nutrients and biotic components (here, as nitrate, plankton and detritus). This approach could offer a valuable addition to ecological science because obtaining validation data for modelling is a serious impediment to research; data are typically so sparse that all too often they are consumed in model testing and configuration, leaving few or none for model validation. If the bulk elemental data were used for optimising the model, then the concurrently collected SIR data could be used for validation. Simultaneously, the approach also tests what we know of organism physiology, decay processes, food quality and quantity relationships, and how we describe all of these within simulation models.

While several researchers (e.g., Nilsen et al. 2008; Van der Lingen and Miller 2011; Deehr et al. 2014) have attempted calibrations of Ecopath models through reference to SIR, that is a rather different approach compared to using SIR data for validation of a dynamic model that is built with (totally coupled to) an explicit dynamic isotope description. Likewise, the use of biovolume spectrum-based analyses to compare with SIR estimates of TL (Basedow et al. 2016) differs in that there is no independent measure of TL.

A systems dynamic simulation, as we used here, does not actually rely on values of TL at all; rather the value of TL is an emergent feature of the functioning of the ecosystem, which is as it should be. Likewise, the organism δ^15^N and whole organism isotope fractionation (Δ^15^N) are also emergent features, stemming from isotope discrimination at key physiological events within each organism functional type. Our work acts as a proof of concept for suggestions that more closely coupled experiments and simulations would help to disentangle isotopic routing of specific molecules and their influence on δ^15^N (and TL) of whole organisms (McCarthy et al. 2007; Wolf et al. 2009).

## Conclusion

Our analysis of SIR signatures vs TLs, and the modelling of them, emphasises that constructing food webs is not simply a matter of connectivity; the dynamics inherent within those connections (including facets of the physiology of the organisms; Dijkstra et al. 2008) are critical. Interactions between these components, the functioning of the abiotic system in which the ecology operates, and changes in those dynamics over the life cycle of organisms collectively explain why SIR cannot robustly describe TLs. Dynamic modelling of organismal elemental and isotope content together, with validation against SIR signatures, offers a powerful unifying approach in ecological research. Only models that are of adequate construction will be able to satisfactorily explain the SIR data. The platform could also be used to generate data series for testing other approaches, such as Bayesian inverse and mixing models.

## Electronic supplementary material

Below is the link to the electronic supplementary material.

Supplementary material 1 (XLSX 27 kb)

Supplementary material 2 (PDF 507 kb)
