# Supplementary material for: Toward a mechanistic understanding of trophic structure: inferences from simulating stable isotope ratios
Source: Mar Biol. 2018 Aug 23;165(9):147. doi: 10.1007/s00227-018-3405-0 (PMC6132504; doi:10.1007/s00227-018-3405-0)
Supplement: Supplementary file 2 — Supplementary material 2 (PDF 507 kb) [file 227_2018_3405_MOESM2_ESM.pdf]

Toward a mechanistic understanding of trophic structure: inferences from simulating stable isotope ratios

Kevin J Flynn[1*], Aditee Mitra[1], Antonio Bode[2]

[1] Biosciences, Swansea University, Singleton Park, Swansea SA2 8PP, UK

[2] Instituto Español de Oceanografía (IEO), Centro Oceanográfico de A Coruña, Apdo. 130, 15080 A Coruña, Spain

* corresponding author; k.j.flynn@swansea.ac.uk; +44 1792 295726;

ORCID 0000-0001-6913-5884

Supplementary information on model construction and operation

Also available are model equations presented in a linear form for placing in a modelling or coding platform; these are given within an Excel file – "*Flynn_et_al Equations.xlsx*".

Base Model Structure

The nitrogen-based model (see Fig. 1) described a 5 level functional type (FT) system, where one FT (Phy) was the primary producer (assumed as non-mixotrophic, and hence assigned TL=1), together with 4 FTs assigned as consumers (here identified as zooplankton Z1 to Z4). The activity of all FTs contributed to a common detrital pool; the death rate of Phy increased with deteriorating N-status thus providing phyto-detritus, while Z1-Z4 contributed to detritus through release of unassimilated (voided) ingestate, as well as via their own death (which increased with deteriorating nutrient status).

To this description of total N, we added a parallel model describing the flow of ^{15}N, with differences in N-specific flow rates accounted for by isotope fractionation. A description and rationale for the SIR and TL calculations are given below; the model code itself is presented in an electronic supplementary material (ESM) file - "*Flynn_et_al Equations.xlsx*". We also ran simulations of a smaller food web (ending at *Z3*, rather than *Z4*); the results were consistent with those shown here.

SIR Calculations

In the following, as an example, the ^{15}N components for consumer (zooplankton) Z1 is described. Isotope discrimination factors are defined for predation (a_Pred), regeneration of ammonium (a_NH4reg), voiding of faecal material (a_Void), mortality (a_Mort), and mixing of water bodies (a_W),

The initial value of the state variable for ^{15}N in Z1 (Z1N15; mg ^{15}N m^{-3}) is defined with reference to the state variable describing total N in Z1 (Z1N; mgN m^{-3}) and a constant defining the initial ratio of ^{15}N:^{14}N (InitR_D15N). Thus:

Z1N15 = Z1N*InitR_D15N/(1+InitR_D15N)

Subsequent flows (mg ^{15}N m^{-3} d^{-1}) into Z1N15 (gain, G_Z1N15) and out of Z1N15 (loss, L_Z1N15) are analogous to flows in and out of Z1 (mgN m^{-3} d^{-1}), but take into account any isotope discrimination.

The value of ^{15}N:^{14}N at the current time-step of biomass for each organism group, and for detritus, are described by variables R_P1, R_Z1, R_Z2 (etc.). Thus, for example, for phytoplankton (P1) the ratio is given by:

R_P1 = P1N15/P1N14

Here, P1N15 is the state variable for ^{15}N in P1, and P1N14 is the ^{14}N content. The ^{14}N content of P1 (P1N14) is given as:

P1N14 = P1N-P1N15

Here, P1N is the state variable value for total N in P1

Ingestion rates (mgN m^{-3} d^{-1}) of each food item in terms of the N-content into consumer Z1 are described by variables UP1_Z1, UZ1_Z1, UZ2_Z1 etc.. This syntax reads as:

“uptake {of food}_{by consumer}”.

The ingestion rate (mg ^{15}N m^{-3} d^{-1}) of each food item in terms of the ^{15}N-content into consumer Z1 are given by analogy through variables UP1_Z1N15, UZ1_Z1N15, UZ2_Z1N15 etc..

Thus, for example, the ^{15}N entry of P1 into Z1is given as:

$$UP1\_Z1N15 = UP1\_Z1/(1/(R\_P1/a\_Pred)+1)$$

The total gain of ^{15}N into Z1N15 (G_Z1N15; mg ^{15}N m^{-3} d^{-1}) is then:

$$G\_Z1N15 = UP1\_Z1N15+UX\_Z1N15+UZ1\_Z1N15+UZ2\_Z1N15+UZ3\_Z1N15+UZ4\_Z1N15$$

The gain of total N into Z1N is G_Z1N, so the ratio ^{15}N:^{14}N of the gain (GR_Z1) is:

$$GR\_Z1 = G\_Z1N15/(G\_Z1N-G\_Z1N15)$$

A proportion of the material ingested (gained) is voided (VX_Z1; mgN m^{-3} d^{-1}). The ^{15}N contribution (VX_Z1N15; mg ^{15}N m^{-3} d^{-1}) is thus given as:

$$VX\_Z1N15 = VX\_Z1/(1/(GR\_Z1/a\_Void)+1)$$

This shows the origins of the calculation, though it simplifies to:

$$VX\_Z1N15 = VX\_Z1/(a\_Void/GR\_Z1+1)$$

We retain the original format below.

Other losses from Z1N15 (all with units of mg ^{15}N m^{-3} d^{-1}) are, by analogy with losses from Z1N, due to respiration contributing to dissolved ammonium (VDA_Z1N15), predation (totPred_Z1N15), mortality (M_Z1N15) and abiotic mixing (W_Z1N15). Thus:

$$VDA\_Z1N15 = VDA\_Z1/(1/(R\_Z1/a\_NH4reg)+1)$$
$$totPred\_Z1N15 = totPred\_Z1/(1/(R\_Z1/a\_Pred)+1)$$
$$mort\_Z1N15 = mort\_Z1/(1/(R\_Z1/a\_Mort)+1)$$
$$W\_Z1N15 = W\_Z1N/(1/(R\_Z1/a\_W)+1)$$

The total loss of ^{15}N from Z1N15 (L_Z1N15; mg ^{15}N m^{-3} d^{-1}) is thus:

$$L\_Z1N15 = W\_Z1N15+VDA\_Z1N15+VX\_Z1N15+totPred\_Z1N15+mort\_Z1N15$$

The value of $\delta^{15}N$ for Z1 (d15_Z1) is given as:

d15_Z1 = 1000*((Z1N15/(Z1N15+Z1N14)-Rstd)/Rstd)

This makes reference to the atmospheric ^{15}N:^{14}N standard value of (Rstd =) 0.0036765.

Trophic Level Calculations

TLs were not apportioned according to the web structures shown in Fig. 1; TLs were computed through reference to the source pathway of the N, which varied over time.

Phy (P1 in the model code, described as a non-mixotrophic primary producer) was confined to TL=1. The status of the other components (Z1, Z2, Z3, Z4, detritus) varied with their prior TL, and with the TL status of the incoming contributing nitrogenous material. The model updated the TL for these components at each time-step.

The trophic level of an organism is a function of the biomass origins of its feed +1 (“+1” because we elevate the trophic status with the transfer of feed into the consumer). To define the TL of each consumer we follow the following steps (see also the ESM file describing the model equations: “*Flynn_et_al Equations.xlsx*”).

The example given is for computing the current TL for Z1 (i.e., for TL_Z1).

First we consider the TL and relative contributions of what is coming in. To do that, we take into account the ingestion rates and TL of each prey type into Z1 to define the intermediate variable ITL_Z1 (with units of TL·mgN·m^{-3}·d^{-1}).

ITL_Z1 =
(UP1_Z1*TL_P1+UZ1_Z1*TL_Z1+UZ2_Z1*TL_Z2+UZ3_Z1*TL_Z3++UZ4_Z1*TL_Z4
+UX_Z1*TL_X)

To calculate the average TL, with reference to the total ingestion rate (G_Z1N; mgN·m^{-3}·d^{-1}), and take account of the elevation in TL with assimilation, we define the ingested average consumption TL as:

IavgUTL_Z1 = 1+ITL_Z1/G_Z1N

The unit for this is now just TL (dimensionless).

The TL for the consumer biomass is described as the TL of the existing biomass de facto being diluted with the incoming material. This dilution event, as a N-specific rate (d^{-1}), is defined by:

$$N\_Z1 = G\_Z1N/Z1N*AE\_Z1$$

Note the involvement of the assimilation efficiency (here, AE_Z1) as this defines the proportion of G_Z1N that is retained (not voided directly) by the consumer. The state variable recording the value of the TL (here, TL_Z1), then has an input rate of:

$$TLin\_Z1 = IavgUTL\_Z1*N\_Z1$$

And an output rate of:

$$TLout\_Z1 = TL\_Z1*N\_Z1$$

For the description of the TL of the detrital pool (TL_X) we proceed as follows:

We need to consider the source of additions of N to the detrital pool XN; these are all expressed with units of $mgN \cdot m^{-3} \cdot d^{-1}$ as originating from:

1. (1-AE) × grazing rate for the consumers
2. Mortality of Z, noting that only a proportion (1-fracMort_DA) of this material enters the detrital pool
3. Death of the phytoplankton (P1) which produces phyto-detritus

These additions are all referenced to their respective TLs.

The voided material inherits the TL of the material grazed; this is referenced to ITL_Z (ingested TL into consumer Z); see the example for ITL_Z1, above. The corpses inherit the TL of the respective zooplankton. Together, the contribution to detritus from all the consumers is given thus:

IavgUTL_X =
(ITL_Z1*(1-AE_Z1)+ITL_Z2*(1-AE_Z2)+ITL_Z3*(1-AE_Z3)+ITL_Z4*
(1-AE_Z4)+TL_Z1*mortX_Z1+TL_Z2*mortX_Z2+TL_Z3*mortX_Z3+TL_Z4*
mortX_Z4)/sumUX_Z

Note that the unit of IavgUTL_X collapses to that for TL (i.e., dimensionless) through division by the value of the total summed addition from zooplankton (with unit $mgN \cdot m^{-3} \cdot d^{-1}$) defined as:

sumUX_Z = G_Z1N*(1-AE_Z1)+G_Z2N*(1-AE_Z2)+G_Z3N*(1-AE_Z3)+G_Z4N*(1-AE_Z4)+mortX_Z1+mortX_Z2+mortX_Z3+mortX_Z4

The total average TL, also taking into account addition of material from phytoplankton death (D_P1; $mgN \cdot m^{-3} \cdot d^{-1}$) with its TL of TL_P1, being added to the detrital TL is given by:

TavgTL_X = (IavgUTL_X*sumUX_Z+TL_P1*D_P1)/(sumUX_Z+D_P1)

The "dilution" rate of the TL for detritus is again the N-specific rate of addition of detritus to the detrital pool. That is, the addition is made relative to the current value of XN ($mgN \cdot m^{-3}$). This dilution rate (d^{-1}) is given by:

N_X = (D_P1+sumUX_Z)/XN

And the inputs and outputs to TL_X are, respectively:

TLin_X = TavgTL_X*N_X

TLout_X = TL_X*N_X

Simulations

The model was constructed within Powersim Constructor (now replaced by Powersim Studio 10; www.powersim.com) running under an Euler integration routine with a step size of 0.015625 d (= 22.5min). A spin-up time of 20 days was used. As the systems describe different dynamics, and extinctions of some consumers can occur after prolonged simulation periods, the 100 day sampling periods shown in the plots were selected to represent a period of contrasting community interaction. This 100 day period was sampled with a resolution of 2 days.
